# Supplementary material for: Optogenetically induced cellular habituation in non-neuronal cells
Source: PLoS One. 2020 Jan 17;15(1):e0227230. doi: 10.1371/journal.pone.0227230 (PMC6968872; doi:10.1371/journal.pone.0227230)
Supplement: S1 File — (DOCX) [file pone.0227230.s001.docx]

**Optogenetically induced cellular habituation in non-neuronal cells**

Mattia Bonzanni^1^, Nicolas Rouleau^1^, Michael Levin^2^, David L. Kaplan^1^

**Supplementary Material**

As previously reported (1), we can calculate the Δ value from the experimental data. Briefly:

$\Delta=\frac{R_{\left( n \right)}-R_{\left( n-1 \right)}}{R_{\left( n-1 \right)}-R_{\left( n-2 \right)}} (1)$

where R is the experimental output (either the raw values or the normalized one) and n is the n^th^ trial of the stimulation protocol per each cell. Since the values do not perfectly follow an exponential decay, and thus pose a problem of which events choose to calculate Δ, we fitted *at priori* the data points with an exponential decay fitting avoiding any biased selection of a subset of events. We then used the fitting values at the n^th^ event ($R_{(n)}^{f}$) to calculate Δ. We can thus re-write eq.1 as follows:

$\Delta=\frac{R_{3}^{f}-R_{2}^{f}}{R_{2}^{f}-R_{1}^{f}} (2)$

Considering that the raw output value at the n^th^ event could be expressed as follows:

$$R_{n}={T^{'}}_{n}+{H^{'}}_{\left( ns \right)0}\pm\sigma\sum_{i=0}^{n-1} \Delta^{i}+B (3)$$

where ${T^{'}}_{n}$ represents the translator element, ${H^{'}}_{\left( ns \right)0}$ the habituation element before the stimulation, $\sigma$ the stimulation factor, $\Delta$ the non-stimulation factor and B the background element, we can re-write eq.3 as follows:

$$R_{n}^{f}=A\pm\sigma\sum_{i=0}^{n-1} \Delta^{i} (4)$$

where $R_{n}^{f}$ represent the fitting value of the raw data output at the n^th^ event and A is a collective element defined as follows: $A={T^{'}}_{n}+{H^{'}}_{\left( ns \right)0}+B$. Eq.4 is the equation of a line, in which $R_{n}^{f}$ values are plotted on the y-axis, $\sum_{i=0}^{n-1} \Delta^{i}$ values are plotted on the x-axis; both parameters are calculated from data. It follows that $\sigma$is the slope of the line and A is the intercept on the y-axis. It is important to use the raw values and not the normalized ones to compute the parameters; the latter case does not allow one to calculate neither $\sigma$ nor A, but rather their ratio ($\sigma=\frac{slope}{1-slope}\cdot A$). Applying this strategy, we provide a summary in Table 1 of the results from the group comparisons first described in the Table 2 in the manuscript.

**S1 Table. Calculation of the model variables from the raw data.**

| **Experimental feature** | **Figure** | **Condition** | **Delta (Δ)** | | **Sigma (σ)** | | **A** | |
| --- | --- | --- | --- | --- | --- | --- | --- | --- |
|  |  |  | **Mean** | **S.E.M.** | **Mean** | **S.E.M.** | **Mean** | **S.E.M.** |
| **Frequency** | Fig.2A | 0.5Hz | 0,43 | 0,05 | 2,82 | 0,58 | 12,48 | 1,03 |
|  | Fig.2A | 1Hz | 0,53 | 0,02 | 1,22 | 0,11 | 7,03 | 0,38 |
|  | Fig.2A | 2Hz | 0,67 | 0,01 | 0,95 | 0,07 | 7,47 | 0,39 |
| **Intensity** | Fig.2E | 2V | 0,57 | 0,19 | 0,76 | 0,58 | 5,65 | 1,83 |
|  | Fig.2E | 5V | 0,53 | 0,02 | 1,22 | 0,11 | 7,03 | 0,38 |
| **Native Channels** | Fig.5D | (-)TEA | 0,53 | 0,02 | 1,22 | 0,11 | 7,03 | 0,38 |
|  | Fig.5D | (+)TEA | 0,66 | 0,03 | 0,67 | 0,57 | 4,86 | 2,19 |
| **Frequency transitions** | Fig.4A | First 1Hz | 0,48 | 0,11 | 1,97 | 0,99 | 8,67 | 2,11 |
|  | Fig.4A | Second 1Hz | 0,39 | 0,09 | 1,5E28 | 2,1E28 | 2E28 | 2,8E28 |

| **Experimental feature** | **Figure** | **Condition** | **Delta (Δ)** | | **Sigma (σ)** | | **A** | |
| --- | --- | --- | --- | --- | --- | --- | --- | --- |
|  |  |  | **Mean** | **S.E.M.** | **Mean** | **S.E.M.** | **Mean** | **S.E.M.** |
| **Frequency** | Fig.2A | 0.5Hz | 0,43 | 0,05 | 2,82 | 0,58 | 12,48 | 1,03 |
|  | Fig.2A | 1Hz | 0,53 | 0,02 | 1,22 | 0,11 | 7,03 | 0,38 |
|  | Fig.2A | 2Hz | 0,67 | 0,01 | 0,95 | 0,07 | 7,47 | 0,39 |
| **Intensity** | Fig.2E | 2V | 0,57 | 0,19 | 0,76 | 0,58 | 5,65 | 1,83 |
|  | Fig.2E | 5V | 0,53 | 0,02 | 1,22 | 0,11 | 7,03 | 0,38 |
| **Native Channels** | Fig.5D | (-)TEA | 0,53 | 0,02 | 1,22 | 0,11 | 7,03 | 0,38 |
|  | Fig.5D | (+)TEA | 0,66 | 0,03 | 0,67 | 0,57 | 4,86 | 2,19 |
| **Frequency transitions** | Fig.4A | First 1Hz | 0,48 | 0,11 | 1,97 | 0,99 | 8,67 | 2,11 |
|  | Fig.4A | Second 1Hz | 0,39 | 0,09 | 1,5E28 | 2,1E28 | 2E28 | 2,8E28 |

Each parameter expressed in eq.3 and experimentally derived in eq.4 is related to the stimulation features and/or the nature and composition of the components of the analyzed system. In detail:

- ${T^{'}}_{n}$ is influenced by the nature and composition of the translator element itself (T’) and by the stimulation features (intensity and stimulation time (t_(s)_)).
- ${H^{'}}_{\left( ns \right)0}$ is influenced by the nature and composition of the habituation element (H) and by any previous history of stimulation.
- $\sigma$ is influenced by the stimulation features (intensity and t_(s)_) and by the nature and composition of both H and T’.
- $\Delta$ is influenced by the non-stimulation time (t_(ns)_), namely the resting period between two consecutive stimulation events, and by the nature and composition of H.
- $B$is stimulus-invariant.

We can collectively cluster the variables (T’, H, t_(s)_, intensity, t_(ns)_ and B) and the parameters (A, $\sigma$ and $\Delta$) in a Euler-Venn system as follows:


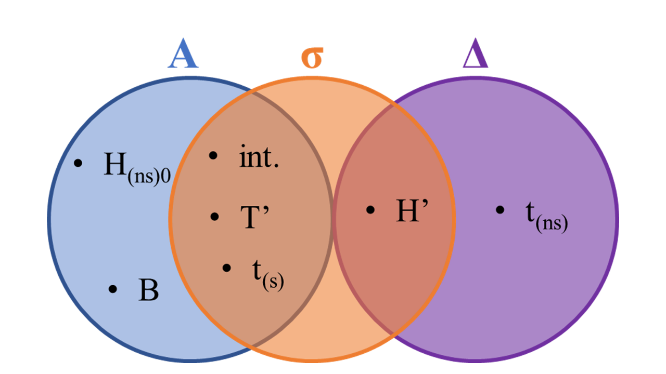


Accordingly, we can compare the calculated parameters (A, $\sigma$ and $\Delta$) among groups and narrow which variables can explain the data. Specifically, we can find that, among groups, the parameters are either significantly different (A; $\sigma$;$\Delta$) or not ($\bar{A}$; $\bar{\sigma}$;$\bar{\Delta}$). We then evaluate the variables that can explain those data. To summarize all the combination, we created Table 1 (main text). Since we control the stimulation during the experimental design, it follows that:

- If, among groups, we have the same stimulation protocol, T’, t_(s)_, t_(ns)_ and intensity are invariant;
- If, among groups, we delivered a novel stimulation (no previous history of stimulation) to the same cell system and the composition of the initial system is unchanged (i.e. no drugs), ${H^{'}}_{\left( ns \right)0}$ is invariant;
- If, among groups, we have the same frequency of stimulation, t_(s)_ and t_(ns)_ are invariant;
- B is always invariant by definition.

Considering the above-mentioned rules and the combinations enlisted in Table 1, we can deduce meaningful insight of some aspect of the mechanism of habituation using an experimental-driven cut off procedures.

**References**

1. Bonzanni M, Rouleau N, Levin M, & Kaplan DL (2019) On the Generalization of Habituation: How Discrete Biological Systems Respond to Repetitive Stimuli: A Novel Model of Habituation That Is Independent of Any Biological System. *Bioessays* 41(7):e1900028.
