# Supplementary figures and images for: Optogenetically induced cellular habituation in non-neuronal cells
Source: PLoS One. 2020 Jan 17;15(1):e0227230. doi: 10.1371/journal.pone.0227230 (PMC6968872; doi:10.1371/journal.pone.0227230)

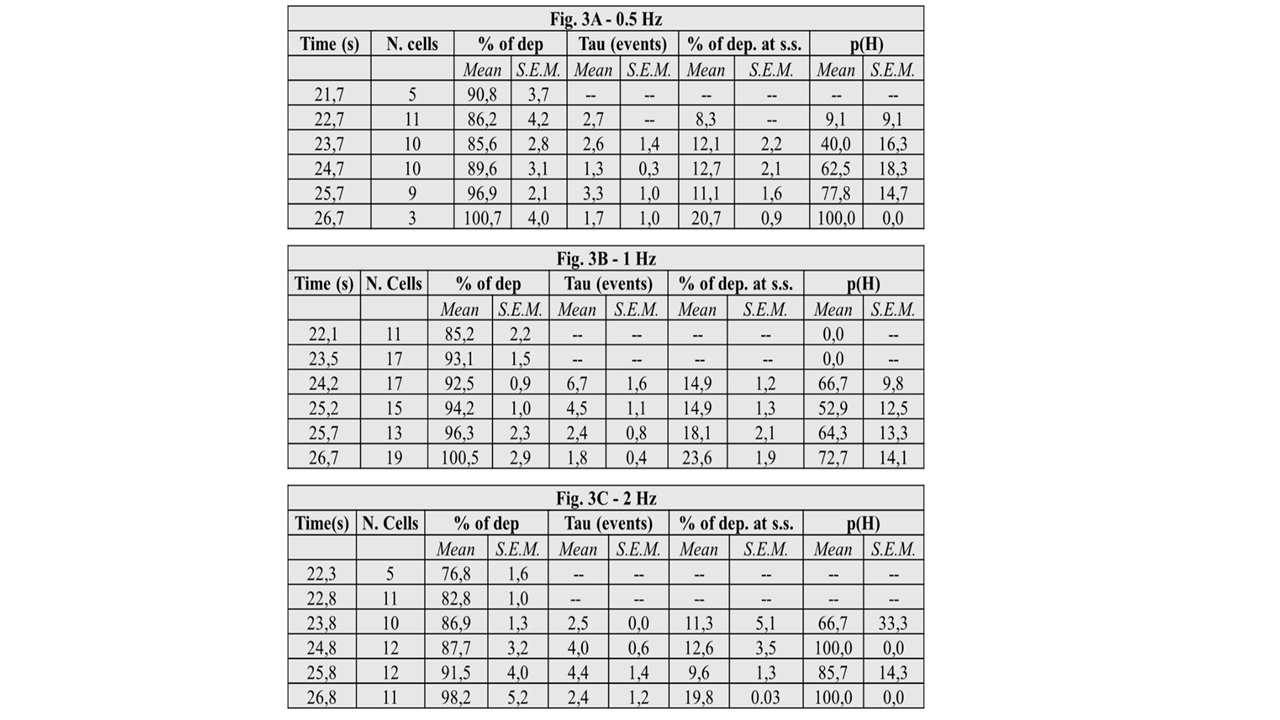

Supplement: S1 Table — (TIF) [file pone.0227230.s006.tif]
