# Supplementary material for: Optogenetically induced cellular habituation in non-neuronal cells
Source: PLoS One. 2020 Jan 17;15(1):e0227230. doi: 10.1371/journal.pone.0227230 (PMC6968872; doi:10.1371/journal.pone.0227230)
Supplement: Attachment — Submitted filename: Mattia Response to Reviewers rev dk final.docx [file pone.0227230.s007.docx]

**December 7, 2019**

**Response to Reviewer Inputs:**

**1) “We note that you have included the phrase “data not shown” in your manuscript. Unfortunately, this does not meet our data sharing requirements.”**

Accordingly, we edited the manuscript. In particular, we added a supplementary figure (Suppl. Fig.3) and removed the sentence.

1) **“Has the statistical analysis been performed appropriately and rigorously? I do not know”**

In order to clarify the performed statistical analysis, we added the following sentences (in red) in the “Statistical Analysis” section:

“Data were analyzed with Clampfit10 (Axon) and Origin Pro 9. To test the impact of the stimulation features on the habituation profile, we compared the mean percentage of depolarization at the steady state and the mean tau of habituation (τH) at different conditions. These two parameters are sufficient to uniquely describe a mono-exponential profile. Data were compared using either One-Way ANOVA followed by Fisher's LSD post-hoc test or Student’s T-test; significance level was set to p=0.05. Data outliers were excluded using Tukey's method. Data were collected from different transfection experiments ranging from a minimum of four to a maximum of twelve.”

2) **“However, it would be great to repeat key experiments of the habituation aspects with the stable cell lines of HEK expressing ChR2 variant. In order to visualize full “Global” cellular response of the optogenetic modulated ionic current on habituation in the non-neuronal cells, authors may consider to perform comparative transcriptomic/ proteomics analysis. This might give a clue about the effect of optogenetically modulated ionic current associated with unknown gene regulation and/ or protein-protein interactions/ protein levels.”**

We thank the reviewer for the comment; as correctly pointed out, our data do not explore the molecular response of the system. Even if it appears to be a limitation in the manuscript, we purposely omitted any molecular investigations. Indeed, the primary aim of the project was to test phenotypic rather than mechanistic similarities between organism-level behavioral and single cell manifestations of habituation. Also at organism level, the molecular pathways are multiple and rarely generalize. As we suggested in the Discussion section, habituation, similarly to positive/negative feedbacks, must be considered a biological strategy to deal with repetitive stimulation and thus studied at the phenotypic level. Even if the reviewer’s suggestions are appropriate, we do feel that they do not apply in the presented cellular model. In this light, we would like to explain our reasons:

- **Conceptual perspective:** As we verbalized in the Discussion section, even if not fully supported by our limited dataset, we do believe that habituations systems do not necessarily share mechanistic processes. It follows that the significance of any molecular insights will be limited to this unique and non-physiological system. It follows that the transcriptomic/proteomics analysis will lack translatability to other systems. As we pointed out in the Limitation Section, the choice of the presented model was dictated by methodological rather than biological considerations. We thus feel that any molecular insight could not be generalized to more relevant systems.
- **Technical perspectives:** Considering the fast onset of the habituation effect (<2 seconds), we are confident that the main effect is due to the modulation of the activity and/or recruitment of the existing repertoire of proteins already present at the time of stimulation. Similarly, in neuronal substrates the short term learning effects are due to either cytosolic events, such as increase of cAMP and phosphorylation events, or the insertion in the membrane of pre-existing pools of proteins (such as AMPA receptors in the early events of LTP). Transcriptional and protein changes occur later and are also associated with a different phenotypic response (short- *vs* long-term effects).
